# Pro-Inflammatory Adipokines as Predictors of Incident Cancers in a Chinese Cohort of Low Obesity Prevalence in Hong Kong

**DOI:** 10.1371/journal.pone.0078594

**Published:** 2013-10-24

**Authors:** Chun-Yip Yeung, Annette Wai-Kwan Tso, Aimin Xu, Yu Wang, Yu-Cho Woo, Tai-Hing Lam, Su-Vui Lo, Carol Ho-Yee Fong, Nelson Ming-Sang Wat, Jean Woo, Bernard Man-Yung Cheung, Karen Siu-Ling Lam

**Affiliations:** 1 Department of Medicine, Li Ka Shing Faculty of Medicine, the University of Hong Kong, Pokfulam, Hong Kong; 2 Research Centre of Heart, Brain, Hormone and Healthy Aging, Li Ka Shing Faculty of Medicine, the University of Hong Kong, Pokfulam, Hong Kong; 3 Department of Pharmacology and Pharmacy, Li Ka Shing Faculty of Medicine, the University of Hong Kong, Pokfulam, Hong Kong; 4 School of Public Health, Li Ka Shing Faculty of Medicine, the University of Hong Kong, Pokfulam, Hong Kong; 5 Department of Strategy and Planning, the Hospital Authority, Kowloon, Hong Kong; 6 Department of Medicine and Therapeutics, The Chinese University of Hong Kong, Shatin, Hong Kong; Geisel School of Medicine at Dartmouth College, United States of America

## Abstract

**Background:**

Cytokines released from adipose tissues induce chronic low-grade inflammation, which may enhance cancer development. We investigated whether indices of obesity and circulating adipokine levels could predict incident cancer risk.

**Materials and Methods:**

This longitudinal community-based study included subjects from the Hong Kong Cardiovascular Risk Factors Prevalence Study (CRISPS) study commenced in 1995-1996 (CRISP-1) with baseline assessments including indices of obesity. Subjects were reassessed in 2000-2004 (CRISPS-2) with measurement of serum levels of adipokines including interleukin-6 (IL-6), soluble tumor necrosis factor receptor 2 (sTNFR2; as a surrogate marker of tumor necrosis factor-α activity), leptin, lipocalin 2, adiponectin and adipocyte-fatty acid binding protein (A-FABP). Incident cancer cases were identified up to 31 December 2011.

**Results:**

205 of 2893 subjects recruited at CRISPS-1 had developed incident cancers. More of the subjects who developed cancers were obese (22.1 vs 16.1%) or had central obesity (36.6 vs 24.5%) according to Asian cut-offs. Waist circumference (adjusted HR 1.02 [1.00-1.03] per cm; p=0.013), but not body mass index (adjusted HR 1.04 [1.00-1.08] per kg/m^2^; p=0.063), was a significant independent predictor for incident cancers after adjustment for age, sex and smoking status. 99 of 1899 subjects reassessed at CRISPS-2 had developed cancers. Subjects who developed cancers had significantly higher level of hsCRP, IL-6, sTNFR2 and lipocalin 2. After adjustment for conventional risk factors, only IL-6 (HR 1.51, 95% CI 1.18-1.95) and sTNFR2 (HR 3.27, 95%CI 1.65-6.47) predicted cancer development.

**Conclusions:**

Our data supported the increased risk of malignancy by chronic low grade inflammation related to central obesity.

## Introduction

The prevalence of obesity has increased dramatically in the last three decades globally[1]. In a World Health Organization report, over 400 million people were estimated to be obese in 2005, and by 2015, the figure will rise to over 700 million[2]. Cardiometabolic disorders related to obesity, including type 2 diabetes, hypertension, dyslipidemia, ischemic heart disease and stroke, cause premature mortality and pose a serious challenge to the modern health care system[1]. In recent years, the association of obesity, in particular central obesity, with cancers has been increasingly recognized[3]. Obesity is associated with adipose tissue inflammation[4] and an increased production of the pro-inflammatory adiopkines such as leptin, interleukin-6(IL-6) and tumor necrosis factor-alpha (TNF-α), lipocalin 2 and adipocyte fatty acid binding protein (A-FABP) which have been shown to enhance tumorigenesis in different tumor cell line models and animal based studies[5,6,7,8] . On the other hand, adiponectin, an anti-inflammatory adipokines with reduced expression in obesity, may be protective against the development of cancers such as breast, endometrial and colorectal cancers[9] . In humans, prospective epidemiological studies have also reported the association of high levels of interleukin-6(IL-6), tumor necrosis factor-alpha (TNF-α) and A-FABP[10,11,12,13], and low level of adiponectin[9], with cancer development. Whether the proinflammatory adipokines are associated with cancer development in the Chinese population, which has a lower prevalence of obesity and different body fat distribution when compared with Caucasians[14,15], remains unclear. In this study, the role of body adiposity and circulating levels of pro-inflammatory adipokines as predictors of incident cancers was addressed in a population-based cohort of Hong Kong Chinese.

## Materials and Methods

### Patients

#### The Hong Kong Cardiovascular Risk Factor Prevalence Study (CRISP) Cohort

The Hong Kong Cardiovascular Risk Factor Prevalence Study Cohort (CRISP) is a population-based prospective study on Chinese, mostly originated from Southern China. The study commenced as a cross-sectional survey in 1995-1996 (CRISP1) [16] with subjects recruited from the general population through random selections of telephone numbers. After baseline assessment, subjects were invited for prospective follow-up to assess the development of major cardiovascular diseases and cancers with subsequent assessments carried out in 2000-2004 (CRISPS2)[17]. Subjects attended all assessments at the Queen Mary Hospital after an overnight fast. At each assessment, demographic data, medical and drug histories, smoking and family histories of cardiovascular diseases and diabetes were obtained using a standardized questionnaire. Subjects were characterized for anthropometric parameters as previously described [16,17]. At CRISPS-2, plasma and serum samples were taken for the measurement of biomarkers including adipokines. Diagnoses of cancers, based on ICD-9 (140-165,170-176,179-209,230-239) and date of diagnosis were verified from the Hong Kong Hospital Authority database, with cut-off date of 31 December 2011. The prospective relationship between incident cancers over 16 years and obesity was assessed using baseline anthropometric parameters measured in 1995-6 (CRISP1). On the other hand, adipokine levels measured in 2000-4 (CRISP2) were used as baseline data in examining the relationship between 9.5-year cancer incidence and obesity-related adipokines. This study was approved by the Institutional Review Board of the University of Hong Kong / Hospital Authority Hong Kong West Cluster, and all participants gave written informed consent.

### Biochemical Measurements

Blood glucose, insulin and lipid levels were measured as described [17,18]. T2DM was defined as fasting glucose ≥126mg/dl and/or 2-hour post-OGTT glucose ≥200mg/dl or already on treatment. Dyslipidemia was defined as having one or more of the following criteria: 1) fasting triglyceride≥150mg/dl; 2) HDL-cholesterol <50mg/dl in females and <40mg/dl in males; 3) LDL-cholesterol >130mg/dl and 4) already on lipid-lowering drugs. HOMA-IR (homeostasis model assessment index) was calculated as fasting glucose (in mmol/L) times fasting insulin (in μIU/ml) divided by 22.5 [19]. High-sensitivity C-reactive protein (hsCRP) was measured with a particle-enhanced immunoturbidimetric assay (Roche Diagnostics, GmbH, Mannheim, Germany) using anti-CRP mouse monoclonal antibodies coupled to latex microparticles. Adiponectin and lipocalin 2 were measured with in-house sandwich ELISA established in our laboratory [18,20]. The other adipokines were measured using commercially available ELISA assay kits- adipocyte-fatty acid binding protein(A-FABP) and leptin: BioVendor Laboratory Medicine, Inc., Modrice, Czech Republic; soluble tumor necrosis factor-alpha receptor-2 (sTNFR2),as a surrogate marker of TNF-α activity: R&D System, Inc., Minneapolis, USA; IL-6: Bender MedSystems GmbH, Vienna, Austria.

### Statistical Methods

All statistical analyses were performed using Statistical Package for the Social Sciences (SPSS Version 16.0; SPSS Inc, Chicago). Baseline variables were compared using chi-square, partial correlation or analysis of variance as appropriate. Variables were tested for normality using Kolmogorox-Smirnov test, and skewed variables were natural-logarithmically transformed before analysis. Biologically relevant variables were entered into Cox proportional hazard regression analyses by forced entry to identify the independent predictors for incident cancers. 

## Results

### Part I. Obesity-related Parameters and Incident Cancers

2895 subjects (1412 men and 1483 women) were recruited at CRISPS-1. Two patients with known history of malignancy were excluded. After a median follow-up of 16.0 years (interquartile range 15.6-16.5 years), 205 patients had developed cancers (Table S1 in File S1) with an incidence rate of 4.62 per 1000 person-years. The commonest type of cancer was carcinoma of the lung (44 subjects), followed by colorectal cancer (36), breast cancer (20), prostate cancer (18) and cancers of the female reproductive tract (14). The median time for cancer development from baseline was 8.7 years (interquartile range 4.9 to 13.2 years). Table 1 shows that, compared to subjects with no incident cancer on follow up, those who developed cancers were more likely to be male (p=0.004), older (p<0.001), current or ex-smoker (p=0.001), have higher body mass index (p=0.002), higher waist circumference (p<0.001, sex-adjusted), and hypertension (systolic blood pressure ≥140 mmHg and/or diastolic blood pressure ≥90 mmHg or on treatment[21]; p<0.001), type 2 diabetes ( p<0.001), and dyslipidemia (p=0.004) at baseline (1995-6; CRISP1). There was no significant difference in HOMA-IR (p=0.119), fasting insulin level (p=0.683) and alcohol use (p=0.678). More of the subjects who developed cancers were obese (p<0.001) or had central obesity (p<0.001) according to Asian cut-offs (BMI≥27.5 kg/m^2^ for obesity and WC≥90cm in male or 80cm in female for central obesity [14,22]). If the general cut-off for obesity (BMI≥30 kg/m^2^) was used, the prevalence of obesity was 8.3% and 6.1% (p=0.197) for cancer and non-cancer group respectively. After adjustment for potential confounding factors, including age, sex, smoking status, WC (adjusted HR 1.02 [1.00-1.03] per cm increase in WC; p=0.013), but not BMI (adjusted HR 1.04 [1.00-1.08] per kg/m^2^ increase in BMI ; p=0.063), was found to be a significant independent predictor for incident cancers (Table 2, Model 1 and Model 2). WC remained as an independent predictor when diabetes status was also taken into consideration. (Model 3 of Table 2; adjusted HR 1.02[1.00-1.03] per cm increase in WC; p=0.019). Analyses were repeated according to estrogen dependency of the tumor types (estrogen dependent tumors included carcinoma of lung, colon, breast, prostate and female reproductive tract[23], which comprised 132 of the 205 cancer cases). WC, but not BMI, had a borderline association with incident cancer after controlling for age, sex and smoking status with or without DM (adjusted HR 1.02 [1.00-1.04]; p=0.067 with DM or HR 1.03[1.00-1.04]; p=0.066 without DM in models).

**Table 1:** Baseline clinical characteristics of CRISPS-1 (1995-6) cohort subjects with and without incident cancers.

	Incident cancer	No cancer	p-value
N	205	2688	--
Sex (% Male)	120 (58.5%)	1290 (48.0%)	**0.004**
Age (years)	56±11.6	45±12.5	**<0.001**
Ever smoker (%)	73 (35.6%)	667 (24.9%)	**0.001**
Alcohol drinker (%)	73 (35.6%)	994 (37.1%)	0.678
Body Mass Index (kg/m^2^)	24.9±3.96	24.1±3.57	**0.002**
Obesity (%)	45 (22.1%)	432 (16.1%)	**0.015**
Waist Circumference (cm)			**<0.001^b^**
Male	86.6±10.2	82.7±9.45	
Female	78.6±9.08	75.0±9.35	
Central obesity (%)	75 (36.6%)	658 (24.5%)	**<0.001**
Hypertension (%)	74 (36.1%)	449 (16.7%)	**<0.001**
Diabetes (%)	36 (18.1%)	241 (9.4%)	**<0.001**
HOMA-IR^a^,^c^	1.21 (0.79-2.11)	1.11 (0.72-1.79)	0.119
Fasting insulin (mIU/L)	5.10 (3.30-7.80)	4.90 (3.20-7.40)	0.683
Dyslipidemia (%)	151 (73.7%)	1707 (63.6%)	**0.004**

Data are expressed as mean±SD unless stated otherwise; ^a^ log-transformed before analysis; ^b^ sex-adjusted p-value; ^c^ excluded 164 subjects with missing data; obesity was defined as BMI ≥27.5kg/m^2^; central obesity was defined as WC>90 cm for men,>80cm for women; ever smoker was defined as current or former smoker; alcohol drinker was defined as self-reported regular consumption of alcohol.

**Table 2:** Cox Proportional hazard regression showing that waist circumference, but not body mass index, independently predicted cancer development.

	*Model 1*			*Model 2*			*Model 3*	
Baseline parameters	HR (95% CI)	p-value		HR (95% CI)	p-value		HR (95% CI)	p-value
Men	1.21 (0.86-1.69)	0.272		1.36 (0.98-1.89)	0.065		1.34 (0.97-1.86)	0.080
Age (years)	1.07 (1.06-1.08)	**<0.001**		1.07 (1.06-1.08)	**<0.001**		1.07 (1.06-1.08)	**<0.001**
WC (cm)	1.02 (1.00-1.03)	**0.013**		--	--		1.02(1.00-1.03)	**0.019**
BMI (kg/m^2^)	--	--		1.04 (1.00-1.08)	0.063		--	--
Diabetes	--	--		--	--		1.20 (0.85-1.68)	0.300
Ever smoker	1.20 (0.86-1.68)	0.295		1.20 (0.86-1.68)	0.287		1.05 (0.96-1.15)	0.313

Ever smoker was defined as current or former smoker; WC, waist circumference; BMI, body mass index.

### Part II. Adipokines and Incident Cancers

1944 subjects returned for CRISPS2 (2000-4) assessment. After excluding 45 subjects with known cancers or incomplete data at CRISP-2, a total of 1899 (882 male and 1017 female) subjects were included in the analysis. 99 had developed cancers after a median follow up interval of 9.5 years (interquartile range 8.8-10.3). Table 3 shows that, compared with subjects with no cancer, those who developed cancers were more likely to be male (p=0.007), older (p<0.001), current or ex-smoker (p=0.03), have higher waist circumference (p=0.014), and type 2 diabetes (p=0.003) and dyslipidemia (p=0.044) at baseline (2000-4; CRISP2). There was no difference in BMI (p=0.189), HOMA-IR (p=0.143), fasting insulin level (p=0.323), proportion with hypertension (p=0.062) and alcohol use (p=0.518). 

**Table 3:** Baseline characteristics of CRISPS-2 (2000-4) cohort subjects with and without incident cancers.

		Incident cancer	No cancer	p-value
N		99	1800	---
Sex (% Men)		59 (59.6)	823 (45.7)	**0.007**
Age (years)		61.2±11.4	51.8±11.8	**<0.001**
BMI (kg/m^2^)		26.6±4.06	24.1±3.51	0.189
Waist circumference (cm)				**0.014^b^**
Men		86.2±9.50	84.2±9.06	
Women		79.0±9.22	76.2±9.10	
Ever smoker (%)		35 (35.4)	459 (25.5)	**0.030**
Alcohol drinker (%)		33 (33.3)	656 (36.5)	0.518
HOMA-IR^a^,^c^		1.85 (1.28-2.89)	1.66 (1.15-2.49)	0.143
Fasting insulin (mIU/L)		7.40 (5.60-11.0)	7.30 (5.20-10.5)	0.323
Diabetes (%)		26 (26.3)	267 (15.0)	**0.003**
Hypertension (%)		35 (35.4)	482 (26.8)	0.062
Dyslipidemia (%)		70 (70.7)	1089 (60.6)	**0.044**

Data are expressed as mean±SD unless stated otherwise; obesity was defined as BMI ≥27.5kg/m^2^; central obesity was defined as WC>90 cm for men,>80cm for women; ^a^ log-transformed before analysis; ^b^ sex-adjusted p-value; ^c^ excluded n=99 subjects on diabetic treatment; Ever smoker was defined as current or former smoker; Alcohol drinker was defined as self-reported regular consumption of alcohol.

Baseline biomarkers in 2000-4 (CRP, IL-6, sTNFR2, leptin, lipocalin 2 and A-FABP) had significantly positive correlation with indices of obesity WC and BMI while adiponectin had significant negative correlation (Table 4). Table 5 shows that subjects who developed cancers had a higher level of baseline hsCRP (p<.001); IL-6 (p<0.001, sex-adjusted); sTNFR2 (p<0.001, sex-adjusted) and lipocalin 2 (p=0.027, sex-adjusted). There was no difference between the two groups in the level of adiponectin, leptin and A-FABP. 

**Table 4:** Partial correlations between baseline obesity parameters and adipokines of CRISPS-2 (2000-4) cohort subjects.

Biomarkers	BMI		WC	
	Sex-adjusted r	p-value	Sex-adjusted r	p-value
CRP	0.38	<0.001	0.37	<0.001
IL-6	0.16	<0.001	0.17	<0.001
sTNFR2	0.11	<0.001	0.17	<0.001
Adiponectin	-0.33	<0.001	-0.30	<0.001
Leptin	0.60	<0.001	0.58	<0.001
Lipocalin 2	0.06	0.009	0.11	<0.001
A-FABP	0.43	<0.001	0.46	<0.001

All biomarkers were log-transformed before analysis.

**Table 5:** Baseline biomarkers of CRISPS-2 (2000-4) cohort subjects with and without incident cancers.

	Incident cancer	No cancer	p-value
N	99	1800	---
*Biomarkers*			
CRP (mg/L)	0.98 (0.50-1.99)	0.72 (0.33-1.55)	**0.001**
IL-6 (pg/ml)			**<0.001**
Men	0.76 (0.53-1.06)	0.58 (0.36-0.89)	
Women	0.65 (0.38-0.98)	0.54 (0.36-0.81)	
sTNFR2 (ng/mL)			**<0.001**
Men	2411.0 (1980.4-2752.6)	1990.4 (1700.1-2368.2)	
Women	2052.4 (1817.2-2787.0)	1815.2 (1562.3-2147.8)	
Adiponectin (mg/ml)			0.150
Men	5.88 (4.41-9.15)	5.50 (3.57-8.61)	
Women	7.90 (4.59-11.92)	7.82 (5.36-11.59)	
Leptin (ng/ml)			0.129
Men	5.50 (2.19-8.76)	4.53 (2.25-7.06)	
Women	13.66 (8.23-21.98)	11.40 (7.98-16.76)	
Lipocalin 2 (ng/ml)			**0.027**
Men	39.04 (30.14-51.73)	37.58 (30.41-47.53)	
Women	34.23 (26.64-57.18)	31.47 (25.32-40.18)	
A-FABP (ng/ml)			0.076
Men	20.90 (15.17-26.66)	19.15(14.09-25.90)	
Women	27.49 (19.14-41.33)	24.24 (17.41-33.36)	

Data are expressed as mean±SD unless stated otherwise; all biomarkers were log-transformed before analysis; all sex-adjusted p-value except for CRP.

After adjustment for age, sex, and smoking status (Model 1 of Table 6), only baseline IL-6 and sTNFR2 remained to be independent predictors for incident cancers with hazard ratio (HR) of 1.51 (95% CI 1.18-1.95, p=0.001) per natural log unit increase in IL-6, and 3.27 (95% CI 1.65-6.47, p=0.001) per natural log unit increase in sTNFR2, respectively. Similar conclusion was reached when WC and/or diabetes status were also included as covariates (Model 2, 3 and 4 of Table 6). Since tumors are possible sources for inflammatory cytokines like IL-6 and TNF-α, analyses were repeated after excluding 8 patients who developed cancers within one year from baseline, to reduce the effect of a preclinical malignancy. Similar results were obtained with baseline IL-6 and sTNFR2 remaining as independent predictors of incident cancers, with HR of 1.53 (1.18-2.00, p=0.002) per natural log unit increase in IL-6, and 3.15 (1.53-6.47, p=0.002) per natural log unit increase in sTNFR2, respectively. 

**Table 6:** Cox proportional hazard regression models showing baseline IL-6 and sTNFR2 independently predicted incident cancers.

	*Model 1*			*Model 2*			*Model 3*			*Model 4*	
	*(Base Model ^b^)*			*(Base Model + WC)*			*(Base Model + Diabetes)*			*(Base Model + WC +Diabetes)*	
Baseline parameters	HR (95% CI)	p-value		HR (95% CI)	p-value		HR (95% CI)	p-value		HR (95% CI)	p-value
CRP, per mg/L^a^	1.20 (0.99-1.44)	0.062		1.17 (0.96-1.42)	0.124		1.19 (0.98-1.43)	0.073		1.17 (0.96-1.42)	0.127
IL-6, per pg/ml^a^	1.51 (1.18-1.95)	**0.001**		1.49 (1.15-1.91)	**0.002**		1.52 (1.18-1.96)	**0.001**		1.50 (1.16-1.94)	**0.002**
sTNFR2, per ng/ml^a^	3.27 (1.65-6.47)	**0.001**		3.21 (1.61-6.39)	**0.001**		3.20 (1.62-6.29)	**0.001**		3.16 (1.59-6.25)	**0.001**
Lipocalin 2, per ng/ml	1.23 (0.79-1.93)	0.363		1.21 (0.77-1.90)	0.401		1.23 (0.78-1.93)	0.367		1.22 (0.77-1.91)	0.397

^a^ Log-transformed before analysis; ^b^ Base Model adjusted for age, sex and smoking status.

## Discussion

There is now increasing evidence that obesity contributes to the development of many cancers, including cancers of colon, breast, liver, gall bladder, endometrium, kidney, prostate and pancreas [24,25]. A 5 kg/m^2^ increase in body mass index (BMI) above normal was found to increase the risk of developing many types of cancers by 24 to 59% in both sexes [3]. Study in Asia-Pacific region also showed a 5-unit increase in BMI above 18.5 kg/m^2^ raised all cancer-mortality by 1.09 times [26]. Although the prevalence of obesity has been shown to be lower in Chinese populations when compared with Western populations even when Asian-Pacific cut-offs for obesity are adopted [15], a rising trend, along with the incidence and mortality of many cancers, is observed and is believed to be related to the westernization of lifestyle[27]. Moreover, body fat composition in Chinese is different from their Caucasian counterparts and Chinese tend to have more central obesity, and thus visceral fat, for a given BMI after adjustment for age and sex [14]. Since the circulating levels of the pro-inflammatory adipokines are higher in the portal vein than in the peripheral arteries in obese subjects [28], visceral fat may play a more important role in contributing to systemic inflammation than the subcutaneous fat. In this Chinese community cohort with a relative low prevalence of obesity (16.5% and 6.2% having BMI ≥27.5 kg/m^2^ and BMI≥30 kg/m^2^ respectively), we demonstrated that waist circumference, an indicator of central adiposity, but not BMI which represents general obesity, was a significant independent predictor of incident cancer development, even after adjusting for the effect of age, the strongest risk factor in this cohort. This differential effect of central and general adiposity on cancer risk has also been demonstrated in other studies in the Asian-Pacific region. In a case-control study of relatively lean Chinese population with mean BMI 21.9 kg/m^2^, an increase in waist-hip ratio increased the risk of prostate cancer by 3 folds while the risk incurred by BMI was not significant[29]. Again, in a meta-analysis of 30 cohort studies in Asian-Pacific region, WC but not BMI, was associated with increased mortality from pancreatic cancer[30]. 

Pro-inflammatory adipokines have been shown in vitro to play significant pathophysiological roles in different stages of tumorigenesis including cancer cell proliferation, angiogenesis and metastasis [6,31] . Apart from their direct effects, adipokines may also interact with sex hormones, sex-hormone binding globulin, insulin-like growth factor or the binding proteins to mediate the development of obesity-related cancers [32]. In this study, we demonstrated the significant correlations between indices of obesity and the baseline levels of pro-inflammatory markers, among which IL-6 and sTNFR2 independently predicted incident cancer development. 

IL-6 and TNF-α are key cytokines involved in inflammation and immunity [33,34]. Both of them had been shown in vitro to be the links between chronic inflammatory states and cancers, by promoting cell proliferation, enhancing angiogenesis and cancer cell metastasis[35,36]. Epidemiological data also supported the role of IL-6 and TNF-α in cancer promotion. In a nested case-control study of 1298 postmenopausal women in the United States, Ho GY et al. demonstrated that subjects with IL-6 in highest quartile had a relative risk of 1.41 of developing colorectal cancer though the association was insignificant after adjustment for baseline insulin level[10]. Heikkila K et al. also showed IL-6 was associated with increased lung and breast cancer risks in a meta-analysis of two prospective cohorts, the British Women’s Heart and Health study and the Caerphilly Cohort[11]. In addition, in a sub-group analysis of the Nurses’ Health Study, Chan et al. showed that the relative risk of development colorectal cancer was 1.67(95% CI 1.05-268, p=0.03) in participants with highest quartile of sTNFR-2 level, when compared with those in the lowest quartile[12]. While our study was the first prospective study examining the effects of the adipokines on cancer development in Chinese population, our findings on IL-6 and sTNFR-2 were in line with those of western cohorts.

Smoking, a well known carcinogenic risk factor, has been associated with level of both IL6 and TNF-α[37,38]. In the current study, smoking was more prevalent at baseline among those who subsequently developed cancers. In the multivariate analysis, however, smoking was not a significant independent risk factor of cancer development, possibly suggesting that the effect of smoking on cancer development is, at least in part, mediated by these proinflammatory cytokines.

The negative findings on adiponectin in this study was unexpected as this anti-inflammatory adipokine inhibited the growth of several breast cancer cell lines in vitro [39]. In epidemiological studies, hypoadiponectinemia had been shown to predict the development of breast, endometrial, colonic, renal cell carcinoma and many hematological malignancies [9]. The association between adiponectin and prostate or lung cancers, on the other hand, was less consistent [40,41]. This might explain why adiponectin did not emerge as a protective factor in our cohort as prostate and lung cancers constituted a significant proportion of our cancer cases. 

This study has several limitations. Firstly, in view of the relatively small numbers of incident cancer cases, the findings of this study should be validated in studies involving larger sample size. Secondly, since baseline assessments were performed more than a decade ago and the list of adipokines is growing, not all adipokines were measured in this study. Thirdly, estradiol and its metabolites might be other important confounding factors which were not measured. Numerous studies have demonstrated the effects of estrogens on the development and progression of different cancers[23] and estrogen production in obesity state will be increased by peripheral aromatization in the adipose tissues. However, our subgroup analyses on obesity parameters and estrogen dependent cancers were limited by small numbers of events, resulting in insignificant power to make a definitive conclusion. Nonetheless, levels of CRP, IL-6, sTNFR2 were found to be higher in subjects with low estrogen levels (post-menopausal females, defined as women aged older than 55, and male subjects) when compared to female subjects aged less than 55 with presumably higher estrogen levels (Table S2 in File S1). Previous studies have also reported a suppressive effect of estrogen on both adipokines in high levels, and at low levels, no effect on IL-6 but stimulatory effect on TNF- α secretion[42]. In obesity, the increased estrogen production by the adipose tissue would be tend to lower the secretion of these adipokines and hence their effect on cancer development. Fourthly, other factors which may impact on cancer development such as family history of malignancy, lifestyle factors like dietary factors and physical activity, and personal history of chronic inflammatory diseases, were not included in the analyses. Furthermore, detailed data on concomitant medications which may impact on inflammation or adipokines levels, such as aspirin, statins, blockers of the renin-angiotensin system and thiazolidinediones, were not recorded. Finally, due to the limitation of an observational study design, our findings could only imply association but not causal relationship, though multiple molecular mechanisms have been identified to link cancer development with elevated cytokines in chronic inflammatory states. 

## Conclusions

In conclusion, we have demonstrated in a population-based study that high waist circumference, and raised serum levels of the pro-inflammatory adipokines, IL-6 and TNFR2, can predict the development of cancers in a Chinese cohort with relatively low obesity prevalence. These associations are supportive of the role of chronic low grade inflammation, related to central obesity, in the development of cancers even though a causal relationship cannot be concluded. When more epidemiological data become available and with a better understanding of the roles of these adipokines in tumourigenesis, risk models can be constructed to identify high risk patients for the development of malignancy so that appropriate measures may be employed to reduce their risks. On the other hand, in patients with established malignancy, the inflammatory cytokines level may act as novel prognostic factors. Furthermore, the inhibition of the inflammatory cytokines provides potential new strategies for the treatment of cancers or for sensitizing tumors to chemotherapeutic agents. While IL-6 and TNF-alpha receptor antagonists are now used clinically for the treatment of a wide range of rheumatological or hematological disorders, it will be of interest to follow-up on patients treated with such agents for any potential beneficial effects on cancer development. 

## Supporting Information

File S1 **Supporting tables.**
(DOCX)
