# Supplementary material for: Pro-Inflammatory Adipokines as Predictors of Incident Cancers in a Chinese Cohort of Low Obesity Prevalence in Hong Kong
Source: PLoS One. 2013 Oct 24;8(10):e78594. doi: 10.1371/journal.pone.0078594 (PMC3813474; doi:10.1371/journal.pone.0078594)
Supplement: File S1 — Supporting tables. (DOCX) [file pone.0078594.s001.docx]

Table S1. Incident Cancer types

| Sites | Number of patients |
| --- | --- |
| Lung | 44 |
| Colon | 36 |
| Breast | 20 |
| Prostate | 18 |
| Female Reproductive tract | 14 |
| Liver | 12 |
| Urinary bladder | 9 |
| Hematological | 8 |
| Nasopharynx | 7 |
| Stomach | 7 |
| Kidney | 6 |
| Pancreas | 6 |
| Biliary tract and gallbladder | 4 |
| Other^a^ | 11 |
| Unknown | 3 |
| Total | 205 |

^a^Brain, skin, soft tissue, esophagus, thyroid, larynx and oral cavity

Table S2. Levels of biomarkers in estrogen positive and estrogen negative states

|  | Low estrogen  [Female older than 55 plus male subjects] | High estrogen  [Female subjects younger than 55] | p-value |  | Low estrogen  [Female subjects older than 55] | High estrogen  [Female subjects younger than 55] | p-value |
| --- | --- | --- | --- | --- | --- | --- | --- |
| N | 1235 | 664 | -- |  | 350 | 664 | -- |
| CRP | 0.81 (0.40-1.72) | 0.58 (0.24-1.24) | <0.001 |  | 1.06 (0.53-2.02) | 0.58 (0.24-1.23) | <0.001 |
| IL-6 | 0.60 (0.39-0.92) | 0.50 (0.34-0.74) | <0.001 |  | 0.62 (0.42-0.93) | 0.50 (0.34-0.74) | <0.001 |
| sTNFR2 | 2034.6 (1721.1-2421.3) | 1713.3 (1494.0-1978.9) | <0.001 |  | 2091.8 (1768.9-2507.0) | 1713.3 (1494.0-1977.8) | <0.001 |

All biomarkers were log-transformed before analysis; data presented as median (interquartile range).
